# Treatment with cinacalcet increases plasma sclerostin concentration in hemodialysis patients with secondary hyperparathyroidism

**DOI:** 10.1186/s12882-016-0392-6

**Published:** 2016-11-15

**Authors:** Piotr Kuczera, Marcin Adamczak, Andrzej Więcek

**Affiliations:** Department of Nephrology, Transplantation and Internal Medicine, Medical University of Silesia, Francuska 20/24 Str, 40-027 Katowice, Poland

**Keywords:** Secondary hyperparathyroidism, Hemodialysis, Cinacalcet, Sclerostin

## Abstract

**Background:**

Sclerostin is a paracrine acting factor, which is expressed in the osteocytes and articular chondrocytes. Sclerostin decreases the osteoblast-related bone formation through the inhibition of the Wnt/β-catenin pathway. Osteocytes also express the Calcium sensing receptor which is a target for cinacalcet. The aim of this study was to assess the influence of six-month cinacalcet treatment on plasma sclerostin concentration in hemodialysed patients with secondary hyperparathyroidism (sHPT).

**Methods:**

In 58 hemodialysed patients with sHPT (PTH > 300 pg/ml) plasma sclerostin and serum PTH, calcium and phosphate concentrations were assessed before the first dose of cinacalcet and after 3 and 6 months of treatment.

**Results:**

Serum PTH concentration decreased after 3 and 6 month of treatment from 1138 (931–1345) pg/ml to 772 (551–992) pg/ml and to 635 (430–839) pg/ml, respectively. Mean serum calcium and phosphate concentrations remained stable. Plasma sclerostin concentration increased after 3 and 6 months of treatment from 1.66 (1.35–1.96) ng/ml, to 1.77 (1.43–2.12) ng/ml and to 1.87 (1.50–2.25) ng/ml, respectively. In 42 patients with cinacalcet induced serum PTH decrease plasma sclerostin concentration increased after 3 and 6 months of treatment from 1.51 (1.19–1.84) ng/ml to 1.59 (1.29–1.89) ng/ml and to 1.75 (1.42–2.01) ng/ml, respectively. Contrary, in the 16 patients without cinacalcet induced serum PTH decrease plasma sclerostin concentration was stable. Plasma sclerostin concentrations correlated inversely with serum PTH concentrations at the baseline and also after 6 months of treatment.

**Conclusions:**

1. In hemodialysed patients with secondary hyperparathyroidism treatment with cinacalcet increases plasma sclerostin concentration 2. This effect seems to be related to decrease of serum PTH concentration.

## Background

Sclerostin is a 22 kDa protein which acts as a soluble inhibitor of the canonical Wnt/β-catenin pathway [1]. It is synthetized mostly by osteocytes as a product of SOST gene. Recently SOST expression has been described not only in the bone and cartilage but also in calcifying arterial tissue and heart valves [2–4], thus linking sclerostin with the vascular calcification. Deletion, or attenuation of SOST gene at the transcriptional level lead to the development of sclerostosis, or van Buchem’s disease – both of these morbidities lead to the increased bone mass [5, 6].

There is growing evidence that the effects of parathyroid hormone (PTH) on bone may be, at least partially, mediated by sclerostin expression. Administration of exogenous PTH leads to the decrease of sclerostin expression in osteocytes of mice [7]. Also in humans an inverse relation between plasma sclerostin and serum PTH concentrations has been found [8].

Chronic kidney disease is associated with the development of secondary hyperparathyroidism (sHPT) and concomitant CKD-MBD (chronic kidney disease-mineral and bone disorders). This leads to the increased incidence of bone fractures, vascular calcification and thus greater morbidity and mortality in patients with CKD.

Plasma concentration of sclerostin tends to increase across the stages of CKD [9, 10] and is significantly elevated in maintenance hemodialysis patients [11]. It is important to stress that it is yet unknown if higher plasma sclerostin is beneficial or harmful in this group of patients. The studies conducted so far considering the influence of sclerostin in the development of arterial calcifications (and atherosclerosis) [12–15] as well as the cardiovascular and total mortality [16–19] yielded conflicting results. There are some studies however linking the increased plasma sclerostin concentrations in CKD with the development of renal osteodystrophy [20].

Calcium sensing receptor (CaSR) is a seven-transmembrane G-protein-coupled receptor, which was classically seen as a mere modulator of the parathyroid hormone (PTH) secretion in the parathyroid glands in response to serum calcium concentration changes [20]. Recently, the expression of CaSR has been documented outside the parathyroid glands, among others in bone [21, 22].

Cinacalcet is a calcimimetic. Such compounds bind to the CaSR leading to its allosteric modulation which results in the increased receptor’s sensitivity to serum calcium [20]. This leads to decreased PTH production in the parathyroid glands and usually concomitant decrease of serum calcium and phosphate concentrations [23–25].

There are more and more lines of evidence suggesting the importance of sclerostin in the etiopathogenesis of the increased morbidity and mortality of maintenance hemodialysis patients. Moreover, there are at least two potential mechanisms of the influence of cinacalcet on plasma sclerostin concentration (direct – via the CaSR activation, and indirect – via the serum PTH concentration decrease). Taking the aforementioned facts into consideration it seemed rational to study the influence of 6-month cinacalcet regimen on plasma sclerostin concentrations in chronic hemodialysis patients with sHPT.

## Methods

Seventy one adult, chronic hemodialysis patients (40 males, 31 females) with sHPT (serum PTH concentration >300 pg/ml) were enrolled in this prospective, open-label, single arm study. Mean age of patients was 53.3 ± 14.8 years, median time of renal replacement therapy was 32 ± 28 months. Exclusion criteria were: age below 18 years, severe liver insufficiency, oversensitivity to any of the study drug compounds, high probability of non-compliance and suspected short life expectancy on renal replacement therapy.

All patients have been dialyzed using bicarbonate dialysate with dialysate ionized calcium concentration of 1.25 mEq/l in all time-points of the study.

All enrolled patients were treated with cinacalcet. Initial dose was 30 mg once daily and was modified, if it was needed, every 4 weeks depending on the serum PTH concentration. The target of treatment was to decrease serum PTH concentration to 150–300 pg/ml. Maximal allowed dose of cinacalcet was 120 mg daily. All patients were cinacalcet naïve and no other parathyroid-suppressive treatment (besides alfacalcidol) has been used during enrollment or previously.

The doses of calcium carbonate and alfacalcidol were flexible in order to avoid hypocalcemia and hypophosphatemia related to the cinacalcet treatment. In some patients aluminum hydroxide was used as a temporary “rescue” therapy in patients with severe hyperphosphatemia.

In every patient plasma sclerostin concentration and serum PTH, calcium, and phosphate concentrations were assessed before the first dose of cinacalcet and then after 3 and 6 months of treatment. Blood samples were collected directly before hemodialysis procedure in the middle of the week. After collection blood samples were centrifuged, serum was aliquoted in 1 ml test-tubes and frozen in -70 °C until assay. Serum PTH concentration was assessed using electrochemiluminescence (ECL) technique (Roche, Mannheim, Germany) and plasma sclerostin concentration was assessed with an ELISA (Teco Medical, San Diego, CA, USA). Intra-assay coefficient of variation for sclerostin was 3.7–4.0 %. Inter-assay coefficient of variation was 4.3–4.8 %.

Serum calcium and phosphate concentration was assessed using the standard procedures of the hospital’s central laboratory (Beckman-Coulter DxC 600 analyzer).

Statistical analyses were performed using the Statistica 10.0 PL software (StatSoft Polska, Cracow, Poland). Shapiro-Wilk test was used to test the variables distribution. Repeated measures ANOVA test with Bonferroni’s correction for multiple comparisons was used to assess the changes of variables over time. One way ANOVA was used to assess the differences between groups and chi^2^ test to assess the differences in qualitative variables. Correlation coefficients were calculated according to the Spearman’s rank correlation method. Additionally, backwards multiple regression models were built (with the plasma sclerostin concentration as a dependent variable and the variables that reached a level of dependence *p* < 0.2 in the univariate regression analyses as independent factors) in each time-point of the study.

Furthermore, similar multiple regression models were built involving only the patients in whom treatment with cinacalcet caused a significant (>10 %) serum PTH concentration decrease.

Results are shown as means with 95 % confidence index (CI), or as means with standard deviation, alternatively as median values with interquartile range (IQR) for variables with skewed distribution. Differences were considered significant when *p* < 0.05.

The study protocol, adherent to Declaration of Helsinki, was approved by the Medical University of Silesia Bioethics Committee (KNW/0022/KB1/56/I/10 - 21.09.2010) and all patients gave their written informed consent for participation in the study.

## Results

From 71 enrolled patients, 58 (35 males, 23 females, mean age 53.8 ± 14.9 years) completed the study. Among 13 patients ruled out of the study 4 people died, 2 received kidney allograft, 2 patients discontinued the study because of permanent decrease of serum PTH concentration below 150 pg/ml, 2 underwent parathyroidectomy, one patient refused to continue the treatment due to paresthesia, one patient withdrew the consent for the study and one moved out and was lost to follow-up.

The mean doses of cinacalcet after 3 and 6 months of treatment were 42 ± 17 mg and 51 ± 23 mg, respectively.

The dosing of calcium carbonate and alfacalcidol was flexible in order to avoid hypocalcemia and hypophosphatemia related to cinacalcet treatment. The mean daily dose of alfacalcidol increased (p for trend = 0.044) from 0.26 μg (0.17–0.36 μg) at the baseline, to 0.33 μg (0.20–0.45 μg) after 3 months and to 0.39 μg (0.25–0.52 μg) after 6 months, but there were no significant differences found between the doses after correction for multiple comparisons. The percentage of patients treated with alfacalcidol did not change significantly [30 (52 %) at the baseline, 34 (54 %) after 3 month of treatment and 40 (69 %) after 6 months; p for trend = 0.06). Moreover, there was a significant (p for trend = 0.01) decrease in the mean dose of aluminum hydroxide (Alusal) from 385 mg/day (170–410 mg/day) at the baseline, to 310 mg/day (80-540 mg/day); *p* = 0.15 after 3 months, and to 180 mg/day (15–350 mg/day); *p* = 0.03, after 6 months of treatment, but the change in the number of patients treated with aluminum hydroxide was not significant [12 (21 %), 8 (14 %) and 5 (8 %) patients, respectively; p for trend = 0.06)]. The mean dose of calcium carbonate (p for trend = 0.055) and the number of patients (p for trend = 0.71) treated with this drug remained stable. The mean dose was 3.49 g/day (2.68–4.30 g/day) at the baseline, 3.84 g/day (2.95–4.72 g/day); *p* = 0.14 after 3 months of treatment 3.88 g/day (3.04–4.72 g/day); *p* = 0.16 after 6 months of treatment. The number of patients treated with calcium carbonate was 53 (91 %), 55 (95 %) and 54 (93 %), respectively.

In patients who completed the study, cinacalcet treatment caused significant decrease of serum PTH concentration after 3 and 6 months of treatment (Table 1). Serum PTH concentration decreased in 42 out of 58 patients (72 %), in the remaining 28 % it increased or remained stable (change of serum PTH < 10 %). The mean decrease of serum PTH concentration after 3 and 6 months of treatment was 32.2 % and 44.2 % respectively.

**Table 1:** Influence of cinacalcet treatment on serum PTH, Scl, calcium and phosphate concentrations in hemodialysed patients with secondary hyperparathyroidism

	Before treatment	After 3 months of treatment	After 6 months of treatment	*p* for trend	*p* vs. baseline (with Bonferroni’s correction)	
					3 months	6 months
Serum PTH concentration [pg/ml]	1138 (931–1345)	772 (551–992)	635 (430–839)	<0.0001	0.0002	<0.0001
Serum calcium concentration [mmol/l]	2.15 (2.07–2.22)	2.11 (2.04–2.17)	2.08 (2–2.15)	0.15	0.86	0.45
Serum phosphate concentration [mmol/l]	2.02 (1.87–2.18)	1.97 (1.81–2.14)	1.90 (1.74–2.05)	0.11	1	0.33
Plasma sclerostin concentration patients with PTH decrease [ng/ml]; (*n* = 42)	1.51 (1.19–1.84)	1.59 (1.29–1.89)	1.75 (1.42–2.01)	0.01	0.74	0.03
Plasma sclerostin concentration patients without PTH decrease [ng/ml]; (*n* = 16)	2.09 (1.32–2.85)	2.33 (1.27–3.40)	2.30 (1.16–3.45)	0.34	0.60	1

*PTH* parathyroid hormone

There were no significant in the mean serum calcium and phosphate concentration during cinacalcet treatment (Table 1).

**Fig. 1:**
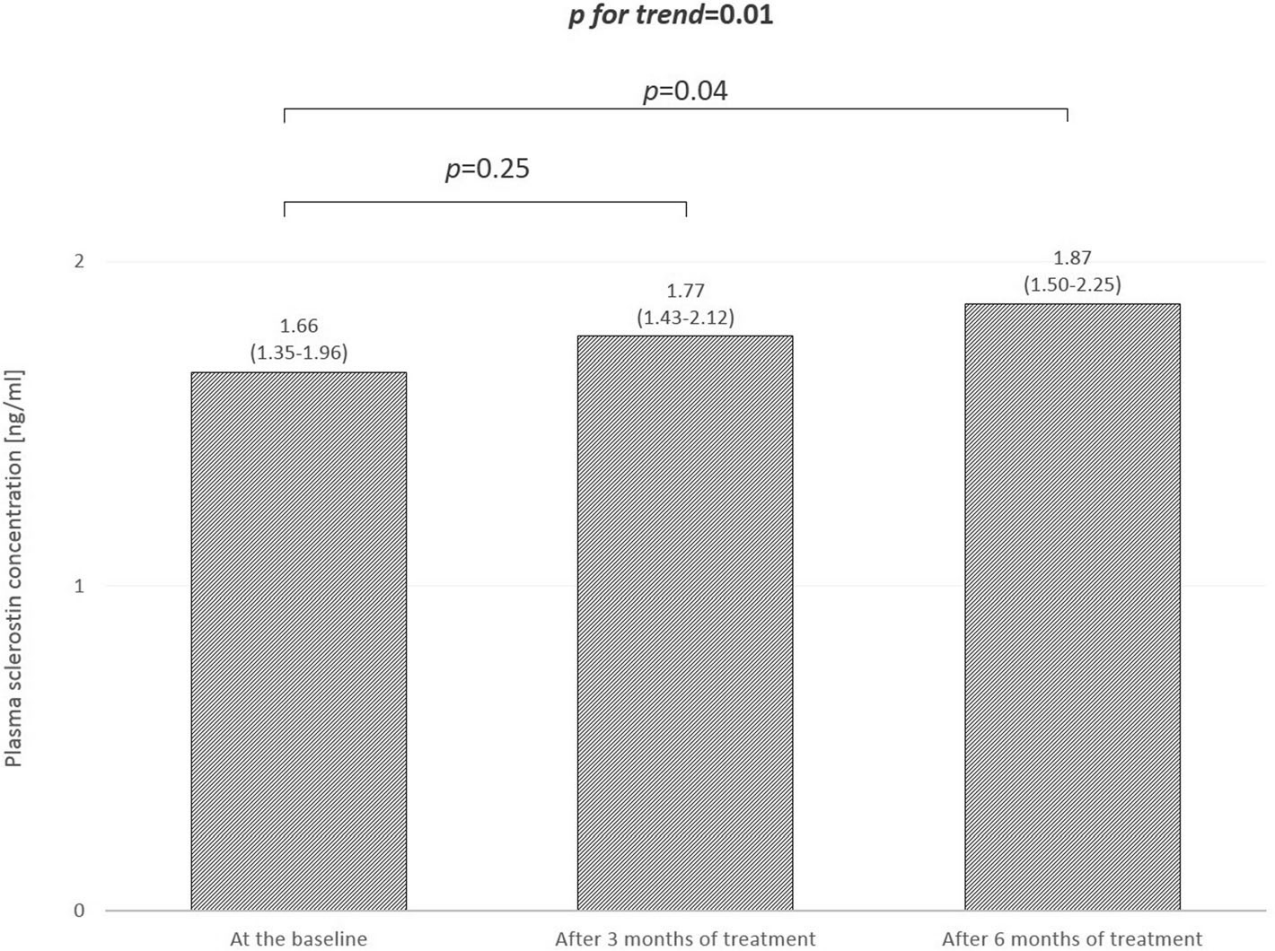
Plasma slerostin concentrations during cinacalcet treatment

Mean plasma sclerostin concentration increased significantly after 6 months of treatment with cinacalcet (Fig. 1).

As it was mentioned above in 42 patients serum PTH concentration decreased after treatment with cinacalcet. In this sub-group plasma sclerostin concentrations also significantly increased. Contrary, in the patients with no serum concentration of PTH decrease, plasma sclerostin concentrations remained stable, but were markedly higher (*p* = 0.045) in comparison with the patients in whom serum PTH concentration decreased (Table 1).

A significant negative correlation between plasma sclerostin and serum PTH concentrations was found at the baseline and after 6 months of treatment (borderline significance after 3 months) – Table 2. Moreover, there was a significant positive correlation between plasma sclerostin and serum phosphate concentrations at the baseline and after 6 months of treatment (borderline significance after 3 months of treatment) – Table 2. There was no significant correlation between plasma sclerostin and serum calcium concentrations or the dose of cinacalcet (Table 2).

**Table 2:** The correlation coefficients between plasma sclerostin concentrations and the serum concentrations of: calcium, phosphate, PTH, and the dose of cinacalcet

		Plasma sclerostin [ng/ml]		
		baseline	3 months	6 months
serum calcium [mmol/l]	baseline	*R* = -0.06; *p* = 0.67		
	3 months		*R* = -0.06; *p* = 0.67	
	6 months			*R* = 0.19; *p* = 0.18
serum phosphate [mmol/l]	baseline	*R* = 0.43; *p* = 0.001		
	3 months		*R* = 0.24; *p* = 0.08	
	6 months			*R* = -0.34; *p* = 0.01
serum PTH [ng/ml]	baseline	*R* = -0.27; *p* = 0.046		
	3 months		*R* = -0.25; *p* = 0.07	
	6 months			*R* = -0.40; *p* = 0.003
cinacalcet dose [mg]	baseline	n/a		
	3 months		*R* = 0.05; *p* = 0.72	
	6 months			*R* = -0.03; *p* = 0.84

*PTH* parathyroid hormone

The magnitude of plasma sclerostin concentration increase was significantly correlated with the change of serum calcium concentration after both 3 and 6 months of treatment, but no correlations were found between the change of plasma sclerostin and the changes of serum concentrations of PTH, phosphate, nor the dose of cinacalcet (Table 3).

**Table 3:** Correlation coefficients between the changes of plasma sclerostin concentration and changes of serum calcium, phosphate and PTH concentration, as well as the dose of cinacalcet after 3 and 6 months of treatment

		Change of plasma sclerostin [ng/ml]	
		0–3 months	0–6 months
change of serum calcium [mmol/l]	0–3 months	*R* = -0.31; *p* = 0.03	
	0–6 months		*R* = -0.37; *p* = 0.009
change of serum phosphate [mmol/l]	0–3 months	*R* = 0.01; *p* = 0.96	
	0–6 months		*R* = -0.06; *p* = 0.67
change of serum PTH [pg/ml]	0–3 months	*R* = -0.07; *p* = 0.64	
	0–6 months		*R* = -0.18; *p* = 0.21
cinacalcet dose [mg]	0–3 months	*R* = -0.13; *p* = 0.36	
	0–6 months		*R* = -0.15; *p* = 0.29

*PTH* parathyroid hormone

To further emphasize the relations between the abovementioned variables, backward multiple regression models were calculated. The results of the multiple regression analyses were similar to those obtained in univariate correlation analyses. Plasma sclerostin concentration at the baseline was explained by the serum concentrations of phosphate (r_partial_ = 0.50; *p* = 0.0002) and PTH (inversely: r_partial_ = -0.38; *p* = 0.005).

After 3 months of treatment plasma sclerostin concentration was explained by the serum concentrations of phosphate (r_partial_ = 0.35; *p* = 0.01) and not PTH (borderline significance: r_partial_ = -0.27; *p* = 0.06). Serum calcium concentration and cinacalcet dose did not retain in the regression model.

After 6 months of treatment plasma sclerostin concentration was explained by the serum concentrations of phosphate (r_partial_ = 0.40; *p* = 0.004) and PTH (inversely: r_partial_ = -0.28; *p* = 0.05). Serum calcium concentration and cinacalcet dose did not retain in the regression model.

Additionally, we have repeated the abovementioned calculations involving only the patients with significant decrease of serum PTH concentration during the cinacalcet treatment.

After 3 months of treatment plasma sclerostin concentration (in patients with PTH decrease) was explained by the serum concentrations of PTH (inversely: r_partial_ = -0.43; *p* = 0.008) and the dose of cinacalcet (*r* = 0.30; *p* = 0.04) and not phosphate, nor calcium (did not retain in the regression model).

After 6 months of treatment plasma sclerostin concentration (in patients with PTH decrease) was explained by the serum concentrations of phosphate (r_partial_ = 0.28; *p* = 0.01) and PTH (inversely: r_partial_ = -0.49; *p* = 0.001) and not cinacalct dose (r_partial_ = 0.45; *p* = 0.15). Serum calcium concentration did not retain in the regression model.

Furthermore, we tested if plasma sclerostin was associated with the magnitude of PTH decrease. In a multiple regression model the change of serum PTH was not significantly inversely explained by sclerostin concentration (r_partial_ = -0.23; *p* = 0.1), still it was the only variable related enough to be retained in the regression model (neither cinacalcet dose, nor the concentrations of calcium and phosphate retained).

## Discussion

In the current clinical study a significant increase of plasma sclerostin concentration in chronic hemodialysis patients with sHPT after six-months treatment with cinacalcet was observed (Fig. 1). Plasma sclerostin concentration seems to be related with serum PTH concentration.

As far as we are aware this is the first study describing the impact of cinacalcet treatment on plasma sclerostin concentration. As it was mentioned before we have found a relation between plasma sclerostin and serum PTH concentrations. Similar results have been obtained by other authors [11, 12]. Also the association between plasma concentration of sclerostin and serum phosphate have been previously described [10].

Interestingly, we have found that the increase of plasma sclerostin concentration was only observed in patients with the decrease of serum PTH concentration (*n* = 42), while in the patients with no serum PTH concentration decrease i.e. “resistant” to cinacalcet treatment plasma sclerostin was stable. These results might suggest that the increase of plasma sclerostin in our group of patients was rather secondary to the decrease of serum PTH concentration than to a direct stimulation of osteoblast CaSR in bone; however the latter cannot be excluded based solely on the current study results.

Moreover, we have observed that in patients with serum PTH concentration decrease caused by the cinacalcet treatment, serum PTH concentration was the strongest predictor of plasma sclerostin concentration what additionally strengthens our conclusions.

The clinical significance of this finding is yet not known as there are conflicting results of clinical trials concerning the influence of sclerostin on vascular calcifications and mortality [12–19]. It is possible that sclerostin is expressed in the calcifying arterial wall in order to inhibit vascular calcification and the measured increase is an effect of a spill-over from the vascular wall. This is probable in the light of the secondary analyses of ADVANCE Study [26], in which a slower coronary arteries calcification was observed under cinacalcet regimen in comparison with the conventional treatment. It is tempting to hypothesize that this might be caused by greater sclerostin expression and thus explain our findings. On the other hand in the paper by Quershi et al [27], the authors conclude, that plasma sclerostin levels predict the magnitude of vascular calcification, still it is yet unknown if the increased concentrations of sclerostin are the cause, or effect of the increased calcification of the vasculature.

Interestingly, Behets et al. [28] found that treatment with cinacalcet reduced the elevated bone formation rate/tissue area in maintenance HD patients. If this effect could be mediated by the increased sclerostin expression is so far unknown, but it could be an elegant confirmation of current study results.

All of the abovementioned studies, sometimes yielding conflicting results, additionally underline the necessity of conduction another clinical studies regarding the influence of sHPT treatment on sclerostin and other markers of bone metabolism.

Another phenomenon which needs further investigation is if plasma sclerostin concentration could be the predictive factor of the resistance to cinacalcet treatment. Such a conclusion seems to emerge from the current study results, but it did not reach the sufficient statistical significance in the multiple regression analysis, thus larger group of patients is needed to confirm this hypothesis.

Our study has some limitations. The most important is the lack of placebo controlled patients. Nevertheless, as cinacalcet is nowadays so commonly used in the treatment of sHPT, conducting a placebo-controlled study with this agent rises some significant methodological and ethical issues. Furthermore, it might be hypothesized that the increase of plasma sclerostin concentration reported in the current study is merely due to the seasonal change as it has been reported by Dawson-Hughes et al [29]. This is however unlikely, as our patients were gradually enrolled into the study in a 13-month period, what precludes the impact of seasonal change. Moreover, the above-mentioned study was conducted in healthy subjects and there is no such data available in the maintenance hemodialysis patients population, in which the baseline sclerostin concentrations are already increased [11].

Summing up, we have found that treatment with cinacalcet increases plasma sclerostin concentration in hemodialysed patients with secondary hyperparathyroidism. It is plausible to assume that such an increase is rather caused by the cinacalcet-related serum PTH decrease than by the direct stimulation of CaSR in bone.

## Conclusions

1. In hemodialysed patients with secondary hyperparathyroidism treatment with cinacalcet increases plasma sclerostin concentration 2. This effect seems to be related to decrease of serum PTH concentration.

## Abbreviations

CaSR: Calcium sensing receptor
CKD: Chronic kidney disease
CKD-MBD: Chronic kidney disease-mineral and bone disorders
PTH: Parathyroid hormone
sHPT: secondary hyperparathyroidism
